# Supplementary material for: Emerging magnetism and anomalous Hall effect in iridate–manganite heterostructures
Source: Nat Commun. 2016 Sep 6;7:12721. doi: 10.1038/ncomms12721 (PMC5025866; doi:10.1038/ncomms12721)
Supplement: Supplementary Information — Supplementary Figure 1-4, Supplementary Note 1 and Supplementary References [file ncomms12721-s1.pdf]

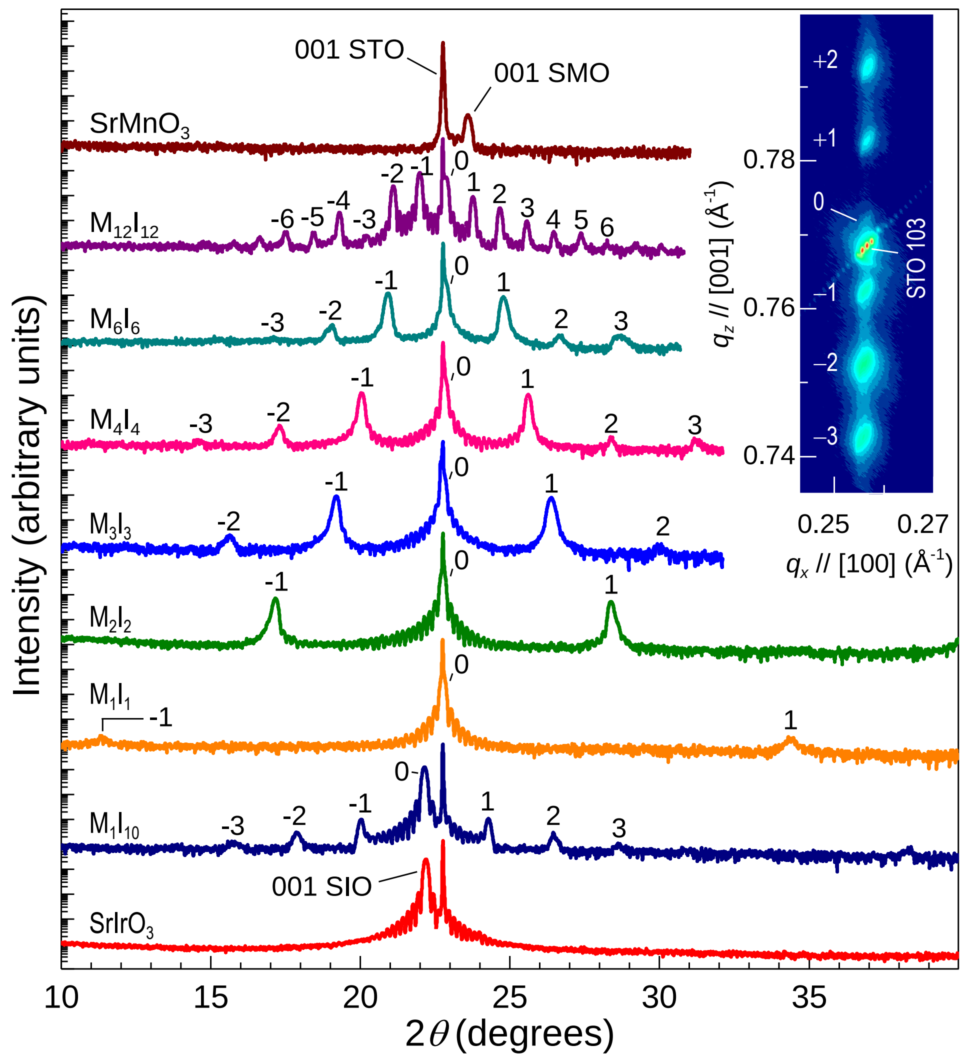

Supplementary Figure 1: Structural properties of SMO-SIO heterostructures. X-ray diffraction θ-2θ scans of $(SrMnO_3)_m/(SrIrO_3)_n$ heterostructures (M_mI_n), $SrMnO_3$, and $SrIrO_3$ films, indicating that all samples are epitaxial, coherently strained, and of very high quality. The inset shows a reciprocal space map near the STO 103 reflection for $M_{12}I_{12}$.

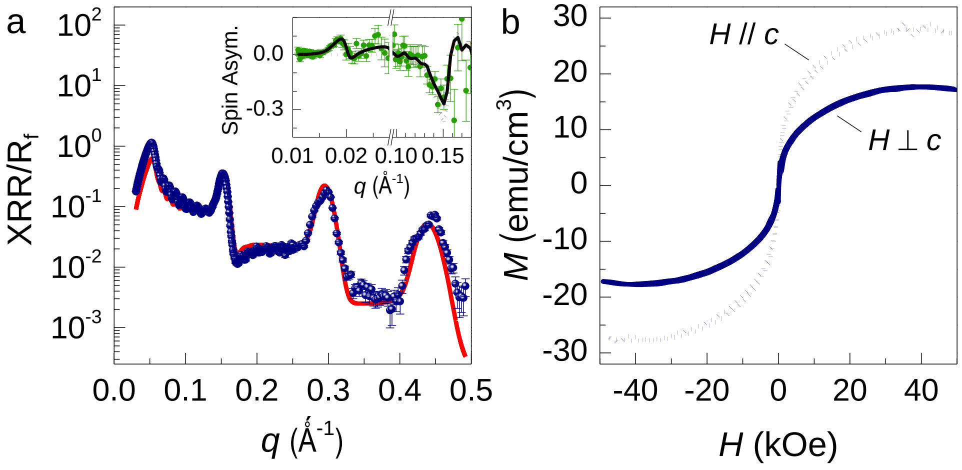

Supplementary Figure 2: Basic characterization of the PNR sample. a, XRR normalized to the asymptotic value of the Fresnel reflectivity (R_f) where the solid line is a fit to the data, which accurately describes the chemical composition of the $[(SrMnO_3)_1/(SrIrO_3)_{10}]_{12}$ sample used for PNR measurements. The inset shows the spin asymmetry where the solid black line represents the ideal fit (M_{Ir} = + 9 emu/cm^3) while the dotted black and dotted red lines represent fits where M_{Ir} is forced to be 0 and – 9 emu/cm^3, respectively. **b,** $M(H)$ of the same sample obtained after a zero field cool at 10K with $H \perp c$ and H // c.

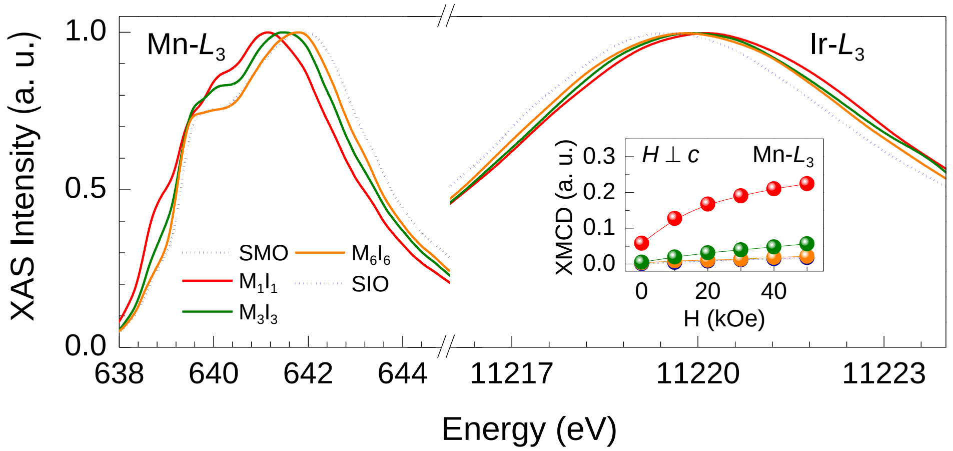

Supplementary Figure 3: XAS and XMCD spectra near Mn and Ir L_3 edges.

Magnification of the XAS spectra from Fig. 2 highlighting the shift of the white line intensity peak. The inset shows the magnetic field dependence of Mn L_3 XMCD peak intensity.

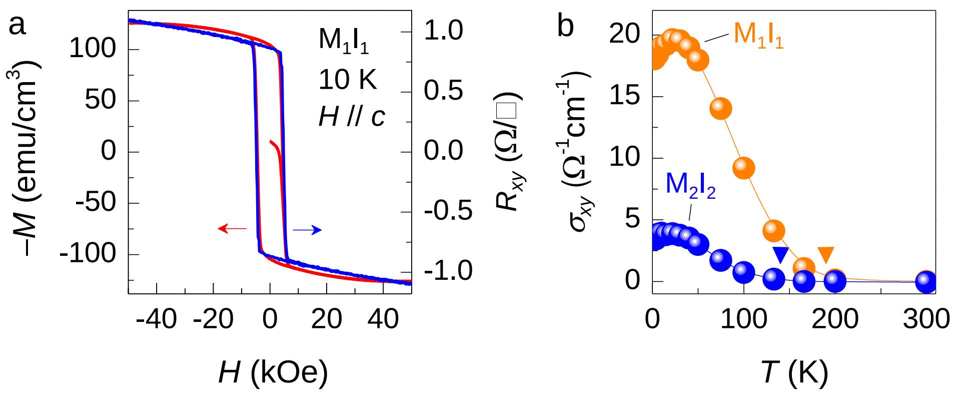

Supplementary Figure 4: Comparison of Hall and SQUID measurements. a, Magnetic field dependence of SQUID (red) and anomalous Hall (blue) data of M_1I_1 at 10 K with *H* // *c*. **b,** Temperature dependence of anomalous Hall conductivity, where the triangles indicate T_c.

Supplementary Note 1

A number of $[(SrMnO_3)_m/(SrIrO_3)_n]_z$ superlattice samples were synthesized by pulsed laser epitaxy on (001) $SrTiO_3$ substrates, where the number of interfaces was varied to maintain an overall sample thickness of roughly 50 nm. Since SIO and SMO have pseudocubic lattice parameters of 3.942 Å[1] and 3.808 Å[2], respectively, SIO is compressively strained, whereas SMO is under large tensile strain on STO (a = 3.905 Å). Bulk SMO is a G-type antiferromagnetic insulator driven by superexchange interactions[3], while SIO is a paramagnetic metal with a J_{eff}=1/2 ground state which is induced by strong spin-orbit coupling [4] that becomes a canted AFI[5,6] under reduced dimensionality[6,7]. The crystal structure, orientation, and phase purity were determined by x-ray diffraction (XRD) measurements. XRD θ-2θ scans near the STO 001 reflection for all samples used in this study are presented in Supplementary Figure 1. We investigated the interface quality through x-ray reflectivity (Supplementary Figure 2a) and conclude that the interfacial roughness is no larger than one unit cell. Combing these results with reciprocal space maps (Supplementary Figure 1) confirm that these samples are epitaxial, have atomically sharp interfaces, are coherently strained, and are of very high quality.

For the Mn L edge data, both electron (surface sensitive) and fluorescence (bulk sensitive) yields were simultaneously monitored and are quite similar indicating that our sample surfaces are not degraded. To better illustrate the change in oxidation state, magnifications of the L_3 edge of both Mn and Ir are presented in Supplementary Figure 3. Additional information regarding the samples used for PNR can be seen in Supplementary Figure 2, where the chemical model (red line) used to simulate the neutron data well describes the chemical composition of the sample to much larger wave vector transfer than can be accessed via neutrons. Also, the $M(H)$ loop clearly

shows the ferromagnetic behavior. We considered the possibility of this interfacial magnetism being influenced by interlayer diffusion. If this were indeed the case, we would expect the overall magnetization (*M*) to scale with the number of interfaces. Since in this study we varied the number of interfaces to maintain a constant sample thickness, *M* should depend inversely on layer thickness (*m*) in that *M* of M_1I_1 should be double that of M_2I_2 and four times that of M_4I_4. However we observe that *M* of M_1I_1 is significantly larger than twice that of M_2I_2 along with M_4I_4 having $M \approx 0$. This result combined with reflectivity data discussed above indicates that the SMO-SIO interfaces are quite sharp without significant chemical intermixing.

Supplementary References

1. Zhao, J. G. et al. High-pressure synthesis of orthorhombic $SrIrO_3$ perovskite and its positive magnetoresistance. *J. Appl. Phys.* **103,** 103706 (2008).
2. Yoshiasa, A., Inoue, Y., Kanamaru, F. & Koto, K. Local structure and spin state of Co^{4+} ions in the perovskite-type $SrCo_{1-x}Mn_xO_3$ solid-solution. *J. Solid State Chem.* **86,** 75-81 (1990).
3. Takeda, T. & Ohara, S. Magnetic-Structure of Cubic Perovskite Type $SrMnO_3$. *J. Phys. Soc. Jpn.* **37,** 275-275 (1974).
4. Kim, B. J. et al. Novel $J_{eff} = 1/2$ Mott state induced by relativistic spin-orbit coupling in Sr_2IrO_4. *Phys. Rev. Lett.* **101,** 076402-076406 (2008).
5. Cao, G., Bolivar, J., McCall, S., Crow, J. E. & Guertin, R. P. Weak ferromagnetism, metal-to-nonmetal transition, and negative differential resistivity in single-crystal Sr_2IrO_4. *Phys. Rev. B* **57,** 11039 (1998).
6. Matsuno, J. et al. Engineering a Spin-Orbital Magnetic Insulator by Tailoring Superlattices. *Phys. Rev. Lett.* **114,** 247209 (2015).
7. Moon, S. J. et al. Dimensionality-Controlled Insulator-Metal Transition and Correlated Metallic State in 5*d* Transition Metal Oxides $Sr_{n+1}Ir_nO_{3n+1}$ (n = 1, 2, and ∞). *Phys. Rev. Lett.* **101,** 226402-226406 (2008).
